# Impact of frailty on postoperative complications in older adults after hip fracture: a systematic review of observational studies

**DOI:** 10.3389/fmed.2025.1667462

**Published:** 2025-11-24

**Authors:** Peng Tian, Yi Yang, Tianjiao He, Liping Wang, Qiurong Zhang, Yingying Cai

**Affiliations:** 1 Department of Geriatrics, The First Affiliated Hospital of Chengdu Medical College, Chengdu Medical College, Chengdu, Sichuan, China; 2 Sichuan Geriatrics Clinical Medical Research Center, Chengdu, Sichuan, China

**Keywords:** frailty, hip fracture, mortality, systematic review, observational studies

## Abstract

**Purpose:**

Frailty, a common syndrome involving multisystem impairment in older adults, is a significant preoperative concern for hip fracture patients. However, its specific link to postoperative complications remains unclear. This systematic review and meta-analysis investigates the association between preoperative frailty and adverse surgical outcomes in this geriatric population.

**Methods:**

We comprehensively searched several databases, including China National Knowledge Infrastructure PubMed, Embase, Web of Science, (China National Knowledge Infrastructure, CNKI), Wanfang, VIP and the Cochrane Library from January 1, 2000, to September 2024. We focused on cohort studies that examine how frailty affects prognosis after hip fracture surgery in older adults. Two researchers independently screened literature, extracted data, and assessed the quality of included studies using the Newcastle-Ottawa Scale (NOS). We used Stata 15.0 to perform the meta-analyses.

**Results:**

This review included 27 cohort studies, consisting of 19 retrospective and 8 prospective studies, involving a total of 243,264 patients. When compared to non-frail patients, frailty statistically significantly increases the risk of postoperative mortality following hip fracture. Specifically, frailty is associated within-hospital mortality [(relative risk, RR) = 3.20, 95% (confidence interval, CI): 1.93, 5.31], 30-day mortality (RR = 3.91, 95%CI: 1.89, 8.07), and 1-year mortality (RR = 1.50, 95%CI: 1.39, 1.61). Frailty also increases the rate of complications (RR = 2.81, 95%CI: 1.67, 4.74), postoperative delirium (RR = 4.44, 95%CI: 2.34, 8.41), pneumonia (RR = 4.09, 95%CI: 2.39, 7.01), and 30-day readmission (RR = 1.75, 95%CI: 1.56, 1.96).

**Conclusion:**

Frailty increases both short-term and long-term mortality following hip fracture surgery in older patients. Additionally, frailty is associated with a higher overall rate of complications, including the 30-day readmission.

## Introduction

Hip fracture, the most prevalent type of fracture among older adults, represents a major global health burden. Projections indicate that the annual number of hip fractures worldwide will reach approximately 6.26 million by 2050, with nearly half of these cases occurring in Asia, underscoring the urgent need for effective management strategies (1, 2). The consequences are severe, with 1-year postoperative mortality rates as high as 36% (3, 4). Although surgery remains the cornerstone of treatment, postoperative complications and readmission rates remain persistently high (5), highlighting a pressing need for reliable risk stratification tools.

In this context, frailty has emerged as a key prognostic factor. Defined as a state of decreased physiological reserve and increased vulnerability, frailty is highly prevalent in older populations and is known to exacerbate adverse outcomes across multiple conditions (6, 7). Its assessment is therefore critical in perioperative management. However, a major challenge lies in the absence of a definitive diagnostic gold standard, which has led to the reliance on a variety of subjective assessment tools in both clinical practice and research, such as the Frailty Phenotype (FP), Clinical Frailty Scale (CFS), and Frailty Index (FI) (8–11). Although these instruments aim to capture the multidimensional nature of frailty, heterogeneity in their components and operational definitions complicates both clinical application and evidence synthesis.

While numerous studies have suggested an association between frailty and poor postoperative outcomes in hip fracture patients (12, 13), the evidence remains inconsistent. Some studies report strong correlations, whereas others have found weak or non-significant associations (14). These discrepancies may stem from differences in the frailty assessment tools used, patient populations, or outcome measures. The 2022 systematic review by Ma et al. provided the first relatively comprehensive synthesis regarding the predictive role of frailty in hip fracture outcomes; however, limited by the number of studies available at the time, the predictive reliability for certain endpoints such as 30-day readmission remained unclear (15). Recent larger-scale clinical studies published in the past 2 years may have altered the predictive landscape for some of these outcomes.

In light of this, we conducted the present systematic review and meta-analysis. Our objectives were not only to synthesize the existing evidence on the association between preoperative frailty and postoperative outcomes in older hip fracture patients but also to perform a comparative analysis of the most commonly used frailty scales. Through this approach, this study aims to provide critical insights to help clinicians select the most appropriate frailty assessment tool, thereby ultimately contributing to improved risk prediction and personalized care for this vulnerable population.

## Materials and methods

### Search strategy

We followed the Preferred Reporting Items for Systematic Reviews and Meta-Analyses (PRISMA) guidelines to report this systematic review. We searched the following databases: CNKI, Wanfang, VIP, CBM, PubMed, Embase, Web of Science, and the Cochrane Library on September 2024. The search employed both Medical Subject Headings (MeSH) and keyword searches. The search terms PubMed include “Fractures, Hip” OR “Intertrochanteric Fractures” OR “Fractures, Intertrochanteric” OR “Trochanteric Fractures” OR “Trochanteric Fractures, Femur” OR “Trochanteric Fracture, Femoral” OR “Trochanteric Fractures, Femoral” OR “Subtrochanteric Fractures” OR “Fractures, Subtrochanteric” AND “Frailties” OR “Frailness” OR “Frailty Syndrome” OR “Debility” OR “Debilities.” Using standardized Excel templates, two investigators independently retrieved data from the included studies; an independent third researcher then cross-checked the extracted data. In cases of disagreement, a third reviewer will arbitrate to reach a consensus.

### Inclusion and exclusion criteria

The inclusion criteria for this systematic review are as follows: (1) Studies published in Chinese or English; (2) Study designs limited to cohort studies, either prospective or retrospective; (3) Study population restricted to older patients with hip fractures, specifically those aged 60 years or older; (4) Research focusing on the impact of preoperative frailty on the prognosis of hip fracture patients; (5) Studies must report at least one of the following outcomes: mortality, postoperative complications such as delirium or pneumonia, and 30-day readmission rates. The exclusion criteria are (1) Studies with incomplete datasets or lacking sufficient statistical analysis; (2) Non-empirical articles, such as commentaries, letters, editorials, conference proceedings, and basic or laboratory-based research; (3) Duplicate publications or multiple reports based on the same dataset, with preference given to the most informative and comprehensive report.

### Data extraction

Two reviewers independently abstracted data from eligible studies using a pre-defined Excel template, with oversight from a third reviewer. Data abstraction included first author, publication year, country of origin, age, study design, sample size, assessment tools, and outcome measures. Mortality was our primary outcome, which included in-hospital mortality, 30-day and 1-year mortality. Secondary outcomes included total complications, postoperative delirium, pneumonia, and 30-day readmission rates.

### Quality assessment

Two reviewers independently assessed the quality of the included cohort studies using the Newcastle-Ottawa Scale (NOS), with verification from a third reviewer. The NOS is a 9-point scale divided into three domains: selection of the study population (0–4 points), comparability of groups (0–2 points), and ascertainment of exposure (0–3 points). Studies with an NOS score of 7 or higher are considered to have high quality.

### Statistical analysis

In this systematic review, we calculated relative risk (RR) and corresponding 95% confidence intervals (CI) for mortality, 30-day readmissions, and postoperative complications. We conducted subgroup analyses to explore variations by study type and frailty assessment instrument. We assessed the heterogeneity of eligible studies using the I^2^ statistic. For I^2^ values of 50% or less, which indicate low heterogeneity, we employed a fixed-effect model in the meta-analysis. For I^2^ values greater than 50%, we used a random-effects model. When we encountered significant heterogeneity, we conducted sensitivity analyses by sequentially excluding each study. We also performed meta-regression to identify potential sources of heterogeneity. We used funnel plot analysis (for meta-analyses including 10 or more studies), Begg’s and Egger’s tests to evaluate the potential publication bias. In our systematic review, we considered a *P* < 0.05 to be statistically significant. We performed all analyses using Stata 15.0.

## Results

### Study screening

**FIGURE 1:**
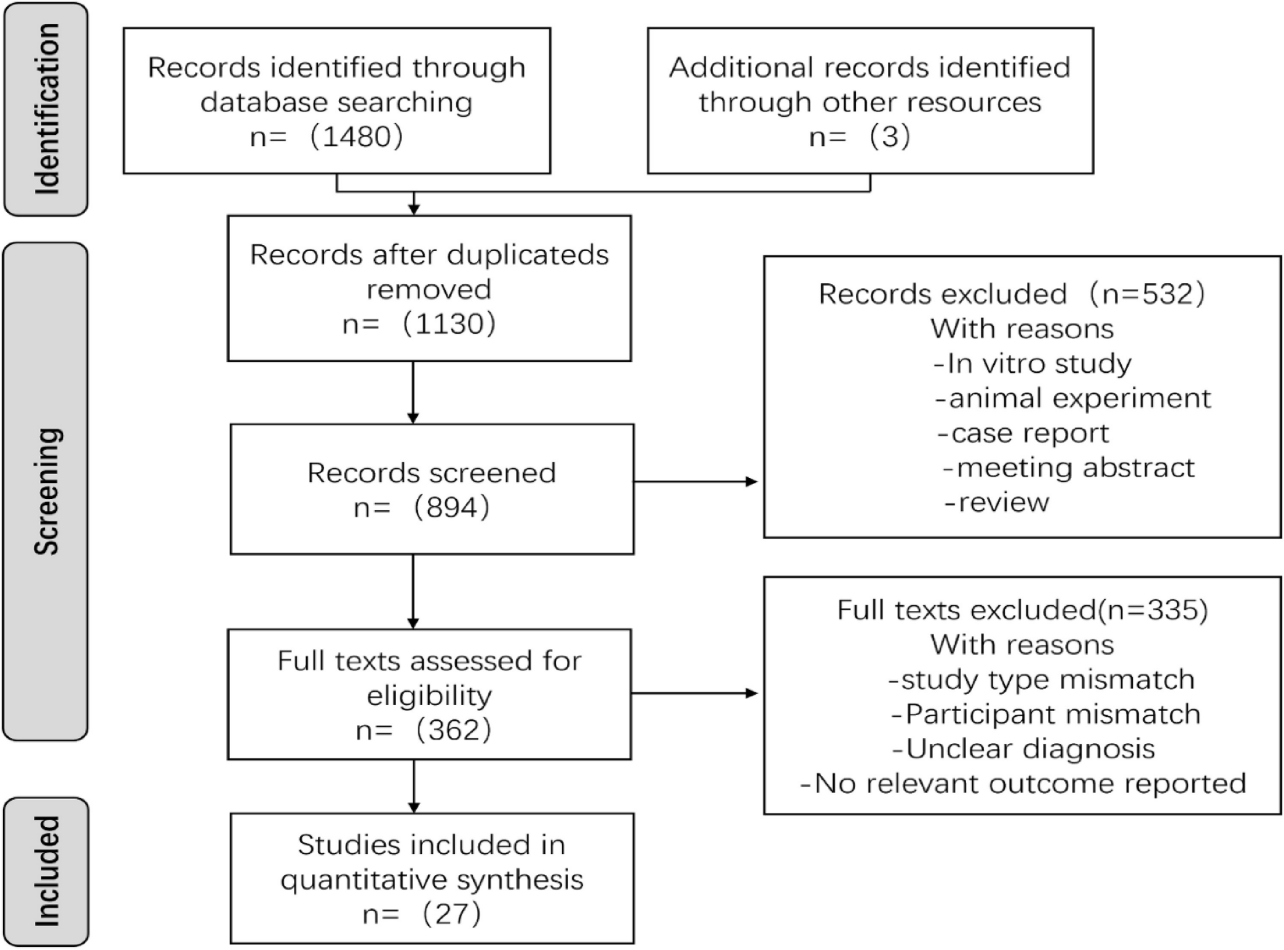
PRISMA flow chart of study screening.

In this study, we identified a total of 1,483 records. After conducting initial abstract and full-text screening, we ultimately included 27 studies, as shown in Figure 1.

### Study characteristics

This systematic review included 243,264 patients across 27 cohort studies (8 prospective and 19 retrospective studies) conducted in 12 countries. Among the 27 studies, 25 were classified as high quality, while 2 were rated as moderate quality, as shown in Table 1. The characteristics of the nine different frailty scales in Table 2.

**TABLE 1:** Characteristics of the included studies.

Author	Year	Country	Age (years)		Study design	Frailty measure	Sample	Quality assessment	Outcomes
Chan et al. (16)	2019	China	≥ 65	82.5 ± 8.4	Retrospective cohort study	Clinical Frailty Scale	423	7	1, 5, 6, 7
Chen e al. (17)	2019	Taiwan, China	≥ 50	78	Prospective cohort study	Chinese-Canadian Study of Health and Aging Clinical Frailty Scale	245	8	2, 4
Winters et al. (18)	2018	Netherlands	≥ 70	83.0 ± 6.6	Prospective cohort study	Veiligheids Management System	277	7	1,2
Kua et al. (15)	2016	Singapore	≥ 60	79.1 ± 9.6	Retrospective cohort study	Reported Edmonton Deal Scale	82	6	5.6.7
Patel et al. (19)	2013	USA	≥ 60	81.05 ± 8.45	Retrospective cohort study	Modified Frail Index	281	7	3
Jorissen et al. (20)	2020	Australia	≥ 65	85.8 ± 6.3	Retrospective cohort study	Frail index	4771	8	2,3
Thorne and Hodgson (21)	2021	UK		85	Retrospective cohort study	Clinical Frailty Scale	1299	6	1,2
Pizzonia et al. (22)	2021	Italy	≥ 65	86.6 ± 5.9	Prospective cohort study	Modified 19-item Frailty Index	364	7	3
Shimizu et al. (23)	2021	Japan	≥ 65	83.6 ± 6.7	Retrospective cohort study	Hospital Frailty Risk Score	31692	7	1,4,6,7
Gandossi et al. (24)	2021	Italy	≥ 65	84.9	Prospective cohort study	Frail index	988	8	6
Gleason et al. (13)	2017	USA	≥ 70	82.3 ± 7.4	Retrospective cohort study	FRAIL Scale	175	8	2,4,5,6,7
Kistler et al. (25)	2015	USA	≥ 65	86.0 ± 4.0	Prospective cohort study	Modified Frail Index	35	7	5, 6, 7
Krishnan et al. (14)	2014	UK		81	Prospective cohort study	Frail Index	178	8	1, 2
Meyer et al. (26)	2020	Germany		65.0 ± 11.4	Retrospective cohort study	Hospital frailty risk Score	4558	7	4, 6, 7
Narula et al. (27)	2020	Australia		82.7 ± 9.1	Retrospective cohort study	Clinical Frailty Scale	509	8	1,2,3
Shen et al. (28)	2021	China	≥ 60	76.7 ± 8.8	Retrospective cohort study	Modified Frail Index	965	7	5, 7
Shin et al. (29)	2016	USA		67.1	Retrospective cohort study	Modified Frail Index	14584	7	2, 5
Wilson et al. (30)	2019	USA	≥ 50	73.7 ± 12.7	Retrospective cohort study	Modified Frail Index	377	8	1, 5
Zhu et al. (31)	2020	China	≥ 65	78.9 ± 6.5	Prospective cohort study	FRAIL Scale	120	8	5,6,7
Pean (32)	2023	USA	≥ 65	78.1 ± 8.1	Retrospective cohort study	Modified Frail Index	9463	8	1,4,5
Walsh et al. (33)	2022	Ireland		80.4 ± 8.8	Retrospective cohort study	Frail index	14615	8	1,6
Gandossi et al. (34)	2023	Italy	≥ 65	84	Prospective cohort study	Frail index	518	8	2, 4,6
Forssten et al. (35)	2023	Sweden	≥ 85	88	Retrospective cohort study	Orthopedic Hip Frailty Score	127305	7	2,
Zhou et al. (36)	2022	China	≥ 60		Retrospective cohort study	modified 5-item Frailty Index	150	7	1,5,6,7
Mitsutake et al. (37)	2024	Australia	≥ 65	83.4 ± 8	Retrospective cohort study	The Hospital Frailty Risk Score	28567	7	1, 4, 5
Choi et al. (38)	2017	Korea	≥ 75.3	80.4	Retrospective cohort study	Hip-MFS(0–14)	481	7	3
Choi et al. (39)	2021	Korea		81.5 ± 6.7	Retrospective cohort study	Hip-MFS(0–14)	242	7	3

Outcomes: 1, in-hosptial mortality; 2, 30-day mortality; 3, 1-year mortality; 4, 30-day readmission; 5, total complications; 6, delirium; 7, pneumonia.

**TABLE 2:** Characteristics of the frailty scales.

Frailty scales	Features	Time to complete	Items	Prediction
Clinical Frailty Scale	Simple and fast; visual and descriptive; holistic; reliable; clinical judgment	5 min	9	In-hosptial mortality; 30-day mortality; 1-year mortality; total complications; delirium; pneumonia
CSHA Clinical Frailty Scale	Pioneering tool; research-backed; global descriptor; validated	2–3 min	7	30-day mortality; 30-day readmission
Veiligheids Management System	Highly simplified, rapid clinical screening tool	2 min	3	In-hosptial mortality; 30-day mortality
Reported Edmonton Deal Scale	Patient-reported; multidimensional; efficient screening tool; highly accessible	5 min	10	Total complications; delirium; pneumonia
Frail Index	Cumulative deficit model; quantitative and continuous; highly flexible; robust predictor; data-driven	10–30 min	20–40	In-hosptial mortality; 30–day mortality; 30-day readmission; delirium
Hospital Frailty Risk Score	Automated and retrospective; uses administrative data; identifies risk, not frailty itself; designed for populations	1 min	109 specific ICD-10 diagnosis codes	30-day readmission; delirium; pneumonia
Hip-MFS	Disease-specific; predictive of outcomes; pre-operative tool; clinical and administrative data	5–10 min	7	1-year mortality
FRAIL Scale	Simple and quick; acronym-based; relies on patient report; strong predictor; screening tool	1–2 min	5	30-day mortality; 30-day readmission; total complications; delirium; pneumonia
Modified Frail Index	Simplified and pragmati; data-driven; strong predictor; no direct assessment needed	2 min	5, 11, 19	In-hosptial mortality; 30-day mortality; 1-year mortality; 30-day readmission; total complications; delirium; pneumonia

#### Mortality

##### In-hospital mortality

**FIGURE 2:**
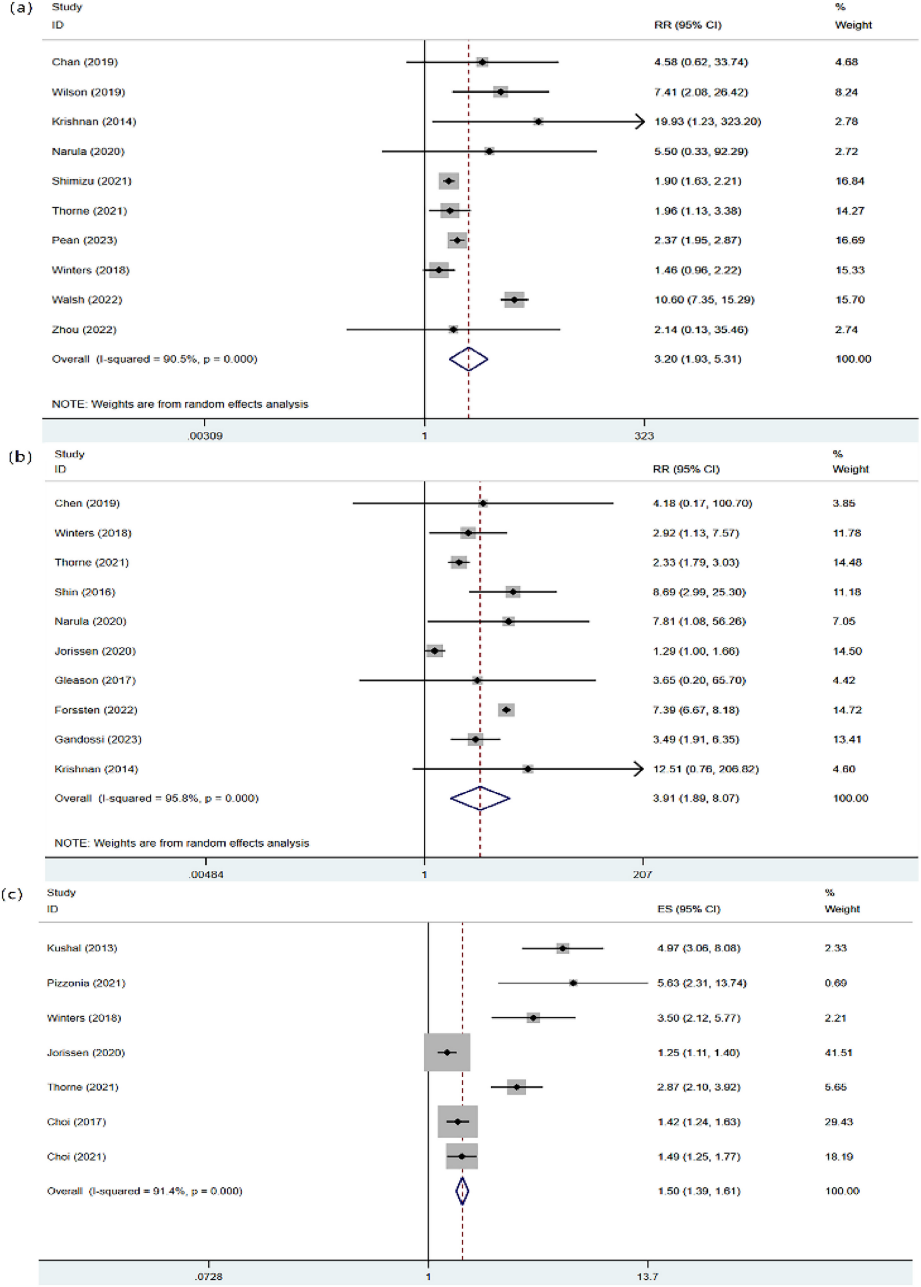
Forest plot of mortality rates in frail patients. **(a)** In-hospital mortality. **(b)** 30-day mortality. **(c)** One-year mortality.

Ten studies involving 61,206 patients examined the impact of preoperative frailty on in-hospital mortality after hip fracture surgery. The meta-analysis revealed high heterogeneity (*I*^2^ = 90.5%); therefore, we used a random-effects model. The results indicated that frailty was statistically significantly associated with in-hospital mortality (RR = 3.20, 95%: CI: 1.93, 5.31), as illustrated in Figure 2.

##### Thirty-day mortality

Ten studies involving 141,164 patients reported the impact of preoperative frailty on 30-day mortality after hip fracture surgery. The meta-analysis revealed high heterogeneity (*I*^2^ = 95.8%). Therefore, a random-effects model was employed, which indicated that preoperative frailty was significantly associated with an increased risk of 30-day mortality (RR = 3.91, 95% CI: 1.89, 8.07), as shown in Figure 2.

##### One-year mortality

Seven studies involving 7,715 patients reported 1-year mortality following hip fracture surgery. The meta-analysis revealed high heterogeneity (I^2^ = 91.4%). It using the random-effects model indicated that preoperative frailty was statistically significantly associated with 1-year mortality (RR = 1.50, 95%CI: 1.39, 1.61), as illustrated in Figure 2.

##### Overall complications

**FIGURE 3:**
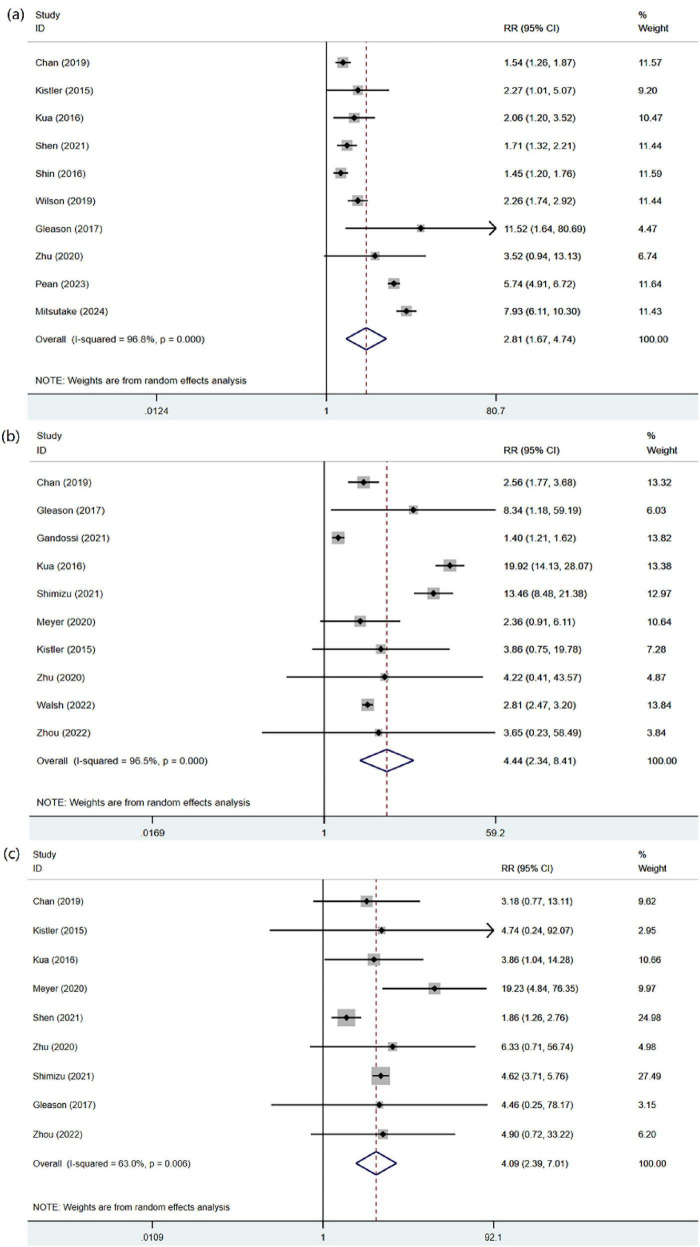
Forest plot of complications in frail patients. **(a)** All complications. **(b)** Deliruim. **(c)** Pneumonia.

A meta-analysis of 10 studies, which included 54,791 patients, was conducted to assess the overall complications following hip fracture surgery with high heterogeneity (*I*^2^ = 96.8%). Frailty significantly increased the risk of complications (RR = 2.81, 95% CI: 1.67, 4.74) in the random-effects model meta-analysis, as shown in Figure 3.

#### Delirium

In a meta-analysis of 10 studies involving 44,537 patients who underwent hip fracture surgery with a high level of heterogeneity (*I*^2^ = 96.5%). Frailty was associated with a significantly increased risk of delirium (RR = 4.44, 95% CI: 2.34, 8.41) in the random-effects model meta-analysis (Figure 3).

#### Pneumonia

Nine studies reported the incidence of postoperative pneumonia in 32,012 patients following hip fracture surgery with moderate heterogeneity (*I*^2^ = 63%). Frailty significantly increased the risk of pneumonia (RR = 4.09, 95% CI: 2.39, 7.01), according to the random-effects model meta-analysis presented in Figure 3.

#### Thirty-day readmission

**FIGURE 4:**
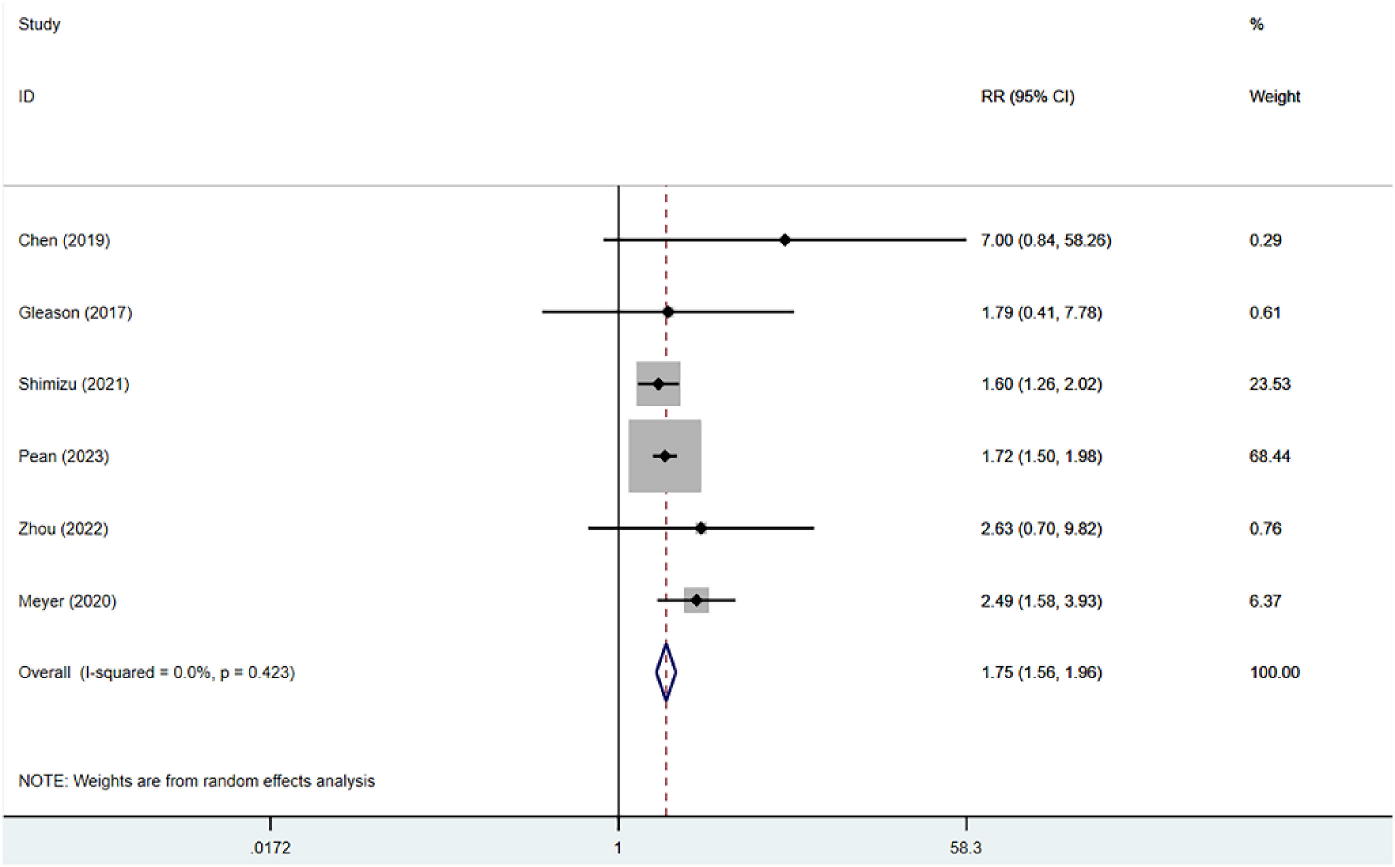
Forest plot of 30-day readmission in frail patients.

Six studies, which included 40,429 patients, reported on 30-day readmissions following hip fracture surgery. The heterogeneity analysis revealed no significant statistical heterogeneity (*I*^2^ = 0%). Frailty was significantly associated with 30-day readmission (RR = 1.75, 95% CI: 1.56, 1.96) in the fixed-effects model meta-analysis, as shown in Figure 4.

#### Sensitivity and subgroup analysis

Sensitivity analysis identified the study by Walsh as the primary source of heterogeneity for in-hospital mortality. Exclusion of this study substantially reduced heterogeneity (*I*^2^ = 40.1%), (RR = 2.08, 95% CI: 1.86, 2.33), though the results remained unstable.

Similarly, for 30-day mortality which exhibited substantial heterogeneity, sensitivity analysis revealed the study by Forssten as the major contributing factor. After removing this study, heterogeneity was reduced (*I*^2^ = 71.9%), (RR = 2.09, 95% CI: 1.70, 2.40), yet the results continued to demonstrate instability.

Subgroup analyses revealed significant variability in results from retrospective studies and certain frailty assessment tools, such as the CFS, demonstrated less heterogeneity. Geographical regions revealed considerable heterogeneity across most outcome measures (Table 3). Meta-regression identified the study design and the frailty assessment tools as the primary sources of this variability.

**TABLE 3:** Subgroup analysis of frailty associated with hip fracture.

Main outcome measure	Numbers	RRs	CIs	I^2^	Design type
In-hosptial mortality	8	3.49	1.97, 6.20	91.9	Retrospective
	2	4.01	0.24, 66.57	76.2	Prospective
30-day mortality	6	3.9	1.53, 9.93	97.6	Retrospective
	4	3.48	2.12, 5.69	0	Prospective
One-year mortality	5	0.38	0.30, 0.45	91.9	Retrospective
	2	1.37	0.93, 1.80	0	Prospective
All complications	8	2.83	1.59, 5.04	97.5	Retrospective
	2	2.56	1.29, 5.08	0	Prospective
Deliruim	7	5.57	2.38, 13.04	96.1	Retrospective
	3	1.62	0.96, 2.72	14	Prospective
Pneumonia	7	4.03	2.23, 7.29	72	Retrospective
	2	5.72	0.98, 33.34	0	Prospective
**Main outcome measure**	**Numbers**	**RRs**	**CIs**	**I^2^**	**Frailty Scales**
In-hosptial mortality	3	3.05	1.47, 6.33	34.1	MFI
	3	2.15	1.28, 3.60	0	CFS
	4	3.76	1.22, 11.60	96.8	Others
30-day mortality	1	8.69	2.99, 25.30	Not applicable	MFI
	2	2.86	1.16, 7.05	31.5	CFS
	7	3.62	1.34, 9.80	96.5	Others
One-year mortality	4	0.44	0.34, 0.55	90.6	MFI
	1	1.05	0.74, 1.37	Not applicable	CFS
	2	0.28	0.16, 0.39	93.6	Others
All complications	5	2.37	1.24, 4.53	97.2	MFI
	1	1.54	1.26, 1.87	Not applicable	CFS
	4	4.65	1.72, 12.59	87.2	Others
Deliruim	4	2.21	1.15, 4.23	94.7	MFI
	1	2.56	1.77, 3.68	Not applicable	CFS
	5	8.91	4.03, 19.70	80.3	Others
Pneumonia	3	1.96	1.34, 2.88	0	MFI
	1	3.18	0.77, 13.11	Not applicable	CFS
	5	4.94	3.62, 6.73	4.1	Others
**Main outcome measure**	**Numbers**	**RRs**	**CIs**	**I^2^**	**Regions**
In-hosptial mortality	2	3.50	1.21, 10.12	67	North America
	4	3.99	1.05, 15.16	95.5	Europe
	3	1.91	1.64, 2.22	0	Asia
	1	5.50	0.33, 92.29	Not applicable	South America and Oceania
30-day mortality	2	7.83	2.87, 21.33	0	North America
	5	3.99	1.87, 8.55	94.6	Europe
	1	4.18	0.17, 100.70	Not applicable	Asia
	2	2.44	0.43, 13.75	69.7	South America and Oceania
One-year mortality	1	1.60	1.12, 2.09	Not applicable	North America
	3	1.16	0.91, 1.41	3	Europe
	2	0.37	0.26, 0.48	0	Asia
	1	0.22	0.11, 0.34	Not applicable	South America and Oceania
All complications	5	3.12	2.81, 3.47	96.9	North America
	–	–	–	–	Europe
	4	1.68	1.44, 1.95	0	Asia
	1	7.93	6.11, 10.30	Not applicable	South America and Oceania
Deliruim	2	5.29	1.51, 18.58	0	North America
	3	2.06	1.10, 3.86	96.4	Europe
	5	7.26	2.18, 24.16	95	Asia
	–	–	–	–	South America and Oceania
Pneumonia	2	4.59	0.58, 36.04	0	North America
	1	19.23	4.84, 76.35	Not applicable	Europe
	6	3.37	1.91, 5.94	69.2	Asia
	–	–	–	–	South America and Oceania

#### Publication bias

The overall risk of publication bias was low; however, funnel plot asymmetry was observed for the specific outcome of 1-year mortality, with both Begg’s test and Egger’s test indicating potential heterogeneity. The trim-and-fill method was subsequently employed to adjust for this bias. Following four iterations, the algorithm imputed two hypothetical studies. After incorporating these studies, bringing the total to nine, the analysis no longer indicated the presence of publication bias. Notably, the adjusted pooled effect size (RR = 6.17, 95% CI: 3.88, 11.36) substantially altered the initial conclusion.

## Discussion

This systematic review demonstrates that hip fracture patients with frailty have a significantly elevated risk of postoperative adverse events compared to their non-frail counterparts. Subgroup analyses further support this conclusion: across different study designs, assessment tools, and geographical regions, outcome indicators including 30-day mortality after discharge, overall complications, postoperative delirium, postoperative pneumonia, and 30-day readmission rates consistently show a stable association between frailty and adverse outcomes.

However, for the specific outcome of in-hospital mortality, different study designs revealed inconsistent strength of association. Retrospective studies indicated that frailty significantly increased the risk of in-hospital mortality, whereas this association did not reach statistical significance in prospective studies. We posit that this discrepancy may be related to the high heterogeneity in frailty assessment tools used across prospective studies (e.g., varied frailty indices, hospital frailty risk scores). Inconsistencies in measurement tools lead to differences in the definition and measurement criteria of key exposure factors; even within the subgroup of higher-quality prospective studies, such heterogeneity may introduce additional variability, thereby diluting the true pooled effect size and reducing statistical power. Furthermore, the analysis of 1-year mortality suggested the presence of publication bias. After adjustment using the trim-and-fill method, the effect size was attenuated and lost statistical significance, indicating that the current evidence for this outcome is limited. In summary, this study supports preoperative frailty as an important predictor of multiple adverse postoperative outcomes in hip fracture patients. However, conclusions regarding specific outcomes such as in-hospital mortality require further validation through more high-quality, prospective cohort studies with standardized measurement tools.

Frailty manifests as diminished physiological reserve and impaired stress response capacity (40), and is closely associated with musculoskeletal disorders. As a serious complication of osteoporosis in older adults (41), hip fracture typically requires surgical intervention, with mortality representing a critical prognostic indicator (42). Previous studies have confirmed that preoperative frailty is significantly associated with in-hospital and post-discharge mortality at various time points among elderly hip fracture patients (43), particularly in the very old population. For instance, an Italian study reported a 1-year postoperative mortality rate of 50% in frail patients, markedly higher than the 10% observed in non-frail counterparts (22). Through this larger-scale systematic review, we further substantiate that preoperative frailty demonstrates strong correlation with 30-day postoperative mortality in hip fracture patients.

Regarding postoperative complications, this systematic review demonstrates a significant association between frailty and the risk of overall postoperative complications. However, substantial heterogeneity was observed in the definition of “overall complications” across studies. While some studies encompassed a broad range of events, including deep vein thrombosis, pneumonia, delirium, and pressure ulcers, others were restricted to only deep vein thrombosis and pneumonia. This inconsistency in definitions, combined with variations in the assessment tools used for frailty, collectively contributed to the heterogeneity in the complication analysis. Sensitivity analysis revealed that the pooled results changed significantly after excluding the studies by Shin and Mitsutake (28, 37). Furthermore, meta-regression confirmed that differences in assessment tools were the primary source of this heterogeneity.

The 30-day readmission rate serves as a crucial quality-of-care indicator (44), with its elevation being closely associated with increased postoperative complication rates. Early readmission following hip fracture surgery not only elevates patient mortality risk but also imposes a substantial financial burden on healthcare systems (45). Furthermore, the study by Ma demonstrated that frailty adversely affects the maintenance of healthy lifespan in older patients, as evidenced by a 1.4-fold higher rate of trauma-related readmissions within 6 months among frail patients compared to their non-frail counterparts (15). Our findings are consistent with this evidence, supporting the clinical value of frailty as a valid predictor for readmission risk.

Heterogeneity analysis revealed substantial heterogeneity across several outcome measures, primarily attributable to highly influential individual studies. Notably, the heterogeneity in in-hospital mortality was mainly driven by the study by Walsh et al., which employed a multidimensional frailty assessment tool and had a large sample size (*n* = 14,625), granting it considerable weight in the pooled analysis. The exclusion of this study resulted in significant changes to the results, indicating limited robustness for this particular outcome. Conversely, heterogeneity in 30-day mortality predominantly originated from the study by Forssten et al. (35), which enrolled an exceptionally large cohort (*n* = 127,305) with a mean age ≥ 85 years, potentially explaining its effect size divergence from the overall trend. These findings suggest that the reliability of the affected outcomes requires further validation through additional high-quality studies with improved methodological consistency.

Comparative assessment of frailty screening instruments demonstrated that although various tools exhibited comparable performance in predicting mortality, complications, and readmission, the choice of instrument remained a major source of heterogeneity. The nine tools examined in this study showed substantial variations in structure and time requirements, ranging from comprehensive multidimensional assessments like the Frailty Index to brief instruments such as the FRAIL scale, with administration times varying from seconds to 60 min. Notably, the Clinical Frailty Scale (CFS) demonstrated particular advantage in predicting in-hospital mortality (46, 47), while both the 5-item modified Frailty Index (mFI-5) and CFS-given their rapid administration, device-independent nature, and minimal training requirements-proved particularly suitable for mobility-restricted hip fracture patients, indicating high clinical feasibility.

Methodologically, this systematic review possesses several methodological strengths as the first to comprehensively evaluate the relationship between frailty and hip fracture outcomes by incorporating both Chinese and English databases. First, we restricted inclusion to cohort studies, thereby effectively minimizing selection and recall biases commonly associated with cross-sectional designs. Second, beyond employing random-effects models for data synthesis and reporting heterogeneity estimates, we conducted sensitivity analyses and meta-regression to trace heterogeneity sources, and performed subgroup analyses based on study design, assessment tools, and geographical regions-collectively demonstrating a comprehensive approach to verifying the robustness of our findings.

Notwithstanding the significantly elevated mortality risk associated with frailty, surgical intervention remains the essential treatment for hip fractures. Based on the accumulated evidence, we recommend implementing either the Clinical Frailty Scale (CFS) or 5-item modified Frailty Index (mFI-5) as standardized preoperative screening tools. Patients identified as frail through these instruments should be enrolled in a systematic perioperative management pathway incorporating prehabilitation interventions, minimally invasive surgical strategies, multimodal analgesia, and targeted postoperative care.

The clinical urgency of this approach is underscored by epidemiological data: frailty prevalence reaches 14.9–31.9% among community-dwelling older adults (48) and is substantially higher in hospitalized populations (49). Moreover, frail individuals face a 1.8-fold increased risk of falls compared to robust counterparts (50). Integrating these epidemiological insights with our findings demonstrating the robust frailty-outcome association, we have identified a critical clinical pathway: “frailty → falls → fracture → postoperative complications.” This cascade represents a significant threat to elderly health.

Therefore, the integration of standardized frailty assessment into routine clinical practice constitutes not only a crucial measure for improving surgical outcomes but also a foundational element in advancing healthy aging. Within future healthcare frameworks, frailty assessment should serve as a cornerstone for risk stratification, systematically informing clinical decision-making across the continuum from community-based prevention to comprehensive perioperative management.

Several limitations of this study should be acknowledged. These include the relatively limited sample sizes of some included studies, absence of detailed data on fracture-specific locations, heterogeneity in frailty assessment tools across studies, and potential publication bias. While this meta-analysis of observational studies can confirm an association, it cannot infer a causal relationship due to potential unmeasured or residual confounding factors. Furthermore, future investigations are warranted with larger-scale prospective designs and standardized assessment protocols to better elucidate the underlying mechanisms through which frailty influences hip fracture outcomes.

## Conclusion

Preoperative frailty is likely to have a substantial impact on the prognosis of hip fracture patients, markedly elevating the risk of patient mortality, postoperative complications, and 30-day readmission rates. The heterogeneity attributable to varying frailty assessment tools is considerable, and future research should prioritize the development of frailty assessment tools tailored for preoperative hip fracture evaluations.

## Data Availability

The original contributions presented in the study are included in the article/supplementary material, further inquiries can be directed to the corresponding author.
